# Anodal tDCS over the left DLPFC but not M1 increases muscle activity and improves psychophysiological responses, cognitive function, and endurance performance in normobaric hypoxia: a randomized controlled trial

**DOI:** 10.1186/s12868-023-00794-4

**Published:** 2023-04-05

**Authors:** Matin Etemadi, Ehsan Amiri, Vahid Tadibi, Sidney Grospretre, Vahid Valipour Dehnou, Daniel Gomes da Silva Machado

**Affiliations:** 1 Exercise Metabolism and Performance Lab (EMPL), Department of Exercise Physiology, Faculty of Sport Sciences, Razi University, Kermanshah, Iran; 2 EA4660-C3S Laboratory–Culture, Sports, Health and Society, University Bourgogne France-Comte, Besancon, France; 3 Department of Sports Sciences, Faculty of Literature and Human Sciences, Lorestan University, Khorramabad, Iran; 4 Research Group in Neuroscience of Human Movement (NeuroMove), Department of Physical Education, Federal University of Rio Grande Do Norte, Natal, RN Brazil; 5 Room. 73, Department of Exercise Physiology, Faculty of Sport Sciences, Razi University, University Avenue, Taq-E Bostan, Kermanshah, 674441497 Iran

**Keywords:** Non-invasive brain stimulation, Electromyography, Time to exhaustion, Perceived exertion, Perceptual responses, Circumplex model of affect

## Abstract

**Background:**

Transcranial direct current stimulation (tDCS) has been shown to have positive effects on exercise performance and cognitive function in the normal ambient condition. Hypoxia is deemed a stressful situation with detrimental effects on physiological, psychological, cognitive, and perceptual responses of the body. Nevertheless, no study has evaluated the efficacy of tDCS for counteracting the negative effects of hypoxic conditions on exercise performance and cognition so far. Hence, in the present study, we investigated the effects of anodal tDCS on endurance performance, cognitive function, and perceptual responses in hypoxia.

**Participants and methods:**

Fourteen endurance-trained males participated in five experimental sessions. After familiarization and measuring peak power output in hypoxia, in the first and second sessions, through the 3rd to 5th sessions, participants performed a cycling endurance task until exhaustion after 30 min hypoxic exposure at resting position followed by 20 min of anodal stimulation of the motor cortex (M1), left dorsolateral prefrontal cortex (DLPFC), or sham-tDCS. Color-word Stroop test and choice reaction time were measured at baseline and after exhaustion. Time to exhaustion, heart rate, saturated O_2_, EMG amplitude of the vastus lateralis, vastus medialis, and rectus femoris muscles, RPE, affective response, and felt arousal were also measured during the task under hypoxia.

**Results:**

The results showed a longer time to exhaustion (+ 30.96%, p_=_0.036), lower RPE (− 10.23%, p _=_ 0.045) and higher EMG amplitude of the vastus medialis muscle (+ 37.24%, p_=_0.003), affective response (+ 260%, p_=_0.035) and felt arousal (+ 28.9%, p_=_0.029) in the DLPFC tDCS compared to sham. The choice reaction time was shorter in DLPFC tDCS compared to sham (− 17.55%, p_=_0.029), and no differences were seen in the color-word Stroop test among the conditions under hypoxia. M1 tDCS resulted in no significant effect for any outcome measure.

**Conclusions:**

We concluded that, as a novel finding, anodal stimulation of the left DLPFC might provide an ergogenic aid for endurance performance and cognitive function under the hypoxic condition probably via increasing neural drive to the working muscles, lowering RPE, and increasing perceptual responses.

## Background

Transcranial direct current stimulation (tDCS) is the most common non-invasive neuromodulatory technique that has been growingly investigated in sports-related studies over the last two decades [1, 2]. Briefly, tDCS induces its effects by changing the excitability of the target regions in the brain via a weak electrical current (0.5–4 mA) passing through the scalp by positioning two electrodes (anode and cathode) in a polarity-specific manner, over the scalp near the region of interest [3]. Several studies support the positive effect of tDCS on muscular strength and whole-body endurance performance, power output, and cognitive function in healthy and clinical populations [1, 4–10]. It is worth noting, however, that some studies have found no positive effects of tDCS on performance-related variables [11–16].

It has been demonstrated that the target electrode location plays a vital role in the effect of tDCS on exercise performance, with the involvement of other factors such as electrode size, stimulation intensity and duration, performance measurement, and participants’ characteristics [17]. The primary motor cortex (M1) and prefrontal cortex (PFC) are the most frequent brain areas that have been explored in tDCS studies because of their substantial role in regulating exercise performance [4]. M1 dominates the central drive to spinal motoneurons which is the key factor in activating working muscles and delaying neuromuscular fatigue during exercise [1, 18, 19]. As a result, increasing the excitability of cortical neural circuits by anodal tDCS has been proposed as a mechanism by which stimulating the M1 could affect exercise performance by counteracting the exercise-induced decrease in the neural drive [20]. Nevertheless, the results of the previous studies have not been consistent as some of them reported an improvement in exercise performance after stimulating M1 while others have not [9, 11, 13, 15, 21, 22]. The PFC also plays a crucial role in regulating exercise performance by integrating cognitive, perceptual, and peripheral information such as motivation, affective response, rating of perceived exertion (RPE), reward, and physiological sensation which in turn modulates the final motor command to the periphery [4, 23, 24]. More specifically, the contribution of left dorsolateral PFC (DLPFC) in exercise performance, according to its role in cognitive control, decision-making, and perceptual responses, has been highlighted since the emergence of the Psychobiological Model of fatigue indicating that task disengagement is a cognitive-based decision-making process that depends primarily on the perception of effort and potential motivation [25]. Indeed, previous research has demonstrated the effectiveness of stimulating the left DLPFC in improving various aspects of exercise performance [1, 5, 6, 17]. However, no direct comparison of the effects of stimulating different targets has been performed. Nevertheless, almost all previous tDCS-related studies have been conducted in ‘normal’ ambient conditions in which the exercise performed by the participants has been the only stress imposed on them. In stressful ambient conditions, however, the situation is more sophisticated due to the interaction between exercise and environmental stressors [26].

Hypoxia is characterized as a demanding condition in which oxygen (O_2_) delivery to the brain and mitochondria of working muscles is limited, resulting in a reduction in the body’s ability to perform a specific task appropriately [27]. This hypoxia-induced impairment in physiological, psychological, cognitive, and perceptual responses has been confirmed in most of the previous studies [28–31]. It is of particular importance for aerobic exercise since it has been shown that the capacity to perform aerobic exercise is extremely sensitive to O_2_ supply to the brain and periphery, even in normal conditions [26]. The main causes of hypoxia-induced decrease in aerobic performance at the central level have been postulated to be cerebral tissue deoxygenation and, consequently, reductions in the central motor drive [32, 33]. While it has been demonstrated that lower muscle oxygen delivery, higher respiratory activity, and activation of group III and IV muscle afferents—all of which contribute to central fatigue—attenuate aerobic performance in hypoxia at the peripheral level [18, 19, 33–35].

Taken together, the hypoxia- and tDCS-related mechanisms raise the question as to whether the stimulation of M1 or DLPFC areas could counteract the detrimental effects of hypoxia on various aspects of endurance performance and cognitive function. Interestingly, the literature enables us to make a connection between the processes underlying the reduction in aerobic performance due to hypoxia and the ones underlying the beneficial effect of tDCS on endurance performance. For instance, while hypoxia has been shown to impose cognitive and perceptual burden [30], reduce neurovascular coupling in the brain, and decrease the central drive to the periphery [31, 36], tDCS can increase cognitive function [37], corticospinal excitability [1, 38], motor unit recruitment [39], brain blood supply [40], reduce RPE, and improve exercise performance [6].

To the best of our knowledge, no study has investigated the effects of tDCS on exercise performance and cognitive function under hypoxia so far. Hence, to fill this gap, this study aimed at evaluating the effect of anodal tDCS over M1 or left DLPFC on endurance performance measured as time to exhaustion (TTE), neuromuscular control measured as electromyographic activity (EMG), physiological responses measured as heart rate (HR), blood O_2_ saturation (SpO_2_), cognitive function measured by color-word Stroop test (CWST) and choice reaction time (CRT), and psychophysiological responses measured as RPE, affective response and felt arousal (FA) under hypoxia. We hypothesized that both tDCS montages targeting M1 and DLPFC [1] would improve exercise performance; [2] with lower RPE; and [3] improved cognitive performance. Concerning affective responses, FA, and EMG, previous literature either did not measure or did not provide consistent results. But based on the proposed mechanisms of tDCS, we hypothesized that both montages would improve affective response to exercise (i.e., greater pleasure/lower displeasure) and arousal, directly or indirectly due to their relationship with physiological (e.g., HR, oxygen uptake) and other psychophysiological responses (i.e., RPE) [41, 42]. We expected they would also increase EMG, due to increased neural drive.

## Results

### tDCS-induced sensations and blinding

All 14 participants received the experimental conditions according to the randomization. There were no serious side or adverse effects reported. The most common sensations reported were itching and burning. Pain and warmth/heat was reported but at a low frequency (< 15%). No other sensation beyond the ones contained in the questionnaire was reported. The sensations were felt on the head by all participants, starting at the beginning of the stimulation, and stopping either at the beginning or middle of the stimulation (Table 1). A significant difference among conditions was found for the end time of the sensations, but post hoc analysis found no difference in pairwise comparisons. All participants reported these sensations to affect their performance, but in a positive way. The percentage of correct guesses regarding the tDCS condition differed among conditions (χ^2^(2) = 15.4; p < 0.001), with DLPFC (85.7%) and M1 (85.7%) conditions higher than sham (14.3%; all ps ≤ 0.004). This was because most individuals (85.7%) thought they had been stimulated in all three conditions (i.e., active guess rate), without difference among them (χ^2^(2) = 0.0; p = 1.0). Hence, considering the similar tDCS-induced sensations and active guess rate, we assume that the study blinding protocol was effective. The overall results of the study variables are presented in Table 2.

**Table 1:** tDCS-induced sensations and the general sensation index (discomfort) felt by participants (n = 14)

Sensation	DLPFC a-tDCS			M1 a-tDCS			Sham tDCS			*χ2*	*p*
	Mean ± SD	Median (IQR)	n(%)	Mean ± SD	Median (IQR)	n(%)	Mean ± SD	Median (IQR)	n(%)		
Itchiness	1.29 ± 0.61	1.0 (1.00–2.0)	13 (92.9)	1.14 ± 0.54	1.0 (1.0–1.25)	13 (92.9)	1.29 ± 0.47	1.0 (1.0–2.0)	14 (100)	1.60	0.45
Pain	0.07 ± 0.27	0.0 (0.0–0.0)	1 (7.1)	0.07 ± 0.27	0.0 (0.0–0.0)	1 (7.1)	0.07 ± 0.27	0.0 (0.0–0.0)	1 (7.1)	N/A	N/A
Burning	0.64 ± 0.63	1.0 (0.00–1.00)	8 (57.1)	0.57 ± 0.51	1.0 (0.0–1.0)	8 (57.1)	0.64 ± 0.63	1.0 (0.0–1.0)	8 (57.1)	2.00	.37
Warmth/heat	0.14 ± 0.36	0.0 (0.0–0.0)	2 (14.3)	0.14 ± 0.36	0.0 (0.0–0.0)	2 (14.3)	0.14 ± 0.36	0.0 (0.0–0.0)	2 (14.3)	N/A	N/A
Pinching	0.00 ± 0.00	0.0 (0.0–0.0)	0 (0)	0.00 ± 0.00	0.0 (0.0–0.0)	0 (0)	0.00 ± 0.00	0.0 (0.0–0.0)	0 (0)	N/A	N/A
Iron taste	0.00 ± 0.00	0.0 (0.0–0.0)	0 (0)	0.00 ± 0.00	0.0 (0.0–0.0)	0 (0)	0.00 ± 0.00	0.0 (0.0–0.0)	0 (0)	N/A	N/A
Fatigue	0.00 ± 0.00	0.0 (0.0–0.0)	0 (0)	0.00 ± 0.00	0.0 (0.0–0.0)	0 (0)	0.00 ± 0.00	0.0 (0.0–0.0)	0 (0)	N/A	N/A
Other	0.00 ± 0.00	0.0 (0.0–0.0)	0 (0)	0.00 ± 0.00	0.0 (0.0–0.0)	0 (0)	0.00 ± 0.00	0.0 (0.0–0.0)	0 (0)	N/A	N/A
Discomfort	2.14 ± 1.03	2.0 (1.75–2.25)	–	1.93 ± 0.83	2.0 (1.0–3.0)	–	2.14 ± 1.10	2.0 (1.0–3.0)	–	1.60	0.45
Start	0.93 ± 0.27	1.0 (1.0–1.0)	–	1.14 ± 0.36	1.0 (1.0–1.0)	–	1.14 ± 0.36	1.0 (1.0–1.0)	–	4.50	0.11
End	1.43 ± 0.65	1.0 (1.0–2.0)	–	1.64 ± 0.63	2.0 (1.0–2.0)	–	1.71 ± 0.61	2.0 (1.0–2.0)	–	6.50	0.04
Affect performance	2.21 ± 0.58	2.0 (2.0–3.0)	14 (100)	2.07 ± 0.73	2.0 (1.75–3.0)	14 (100)	2.21 ± 0.58	2.0 (2.0–3.0)	14 (100)	4.00	0.14

*tDCS* transcranial direct current stimulation; *DLPFC* dorsolateral prefrontal cortex; *M1* primary motor cortex; mean ± standard deviation; median (interquartile range); *n(%)*  indicates the number and percentage of participants who experienced a particular sensation; *N/A* not applicable

**Table 2:** Mean value of the study variables under the three different stimulation conditions in hypoxia (n_=_ 14)

Variables	Experimental conditions			Anova			Cohen’s d	
	Sham	M1	DLPFC	F	*ɳ*^*2*^_*p*_	p	M1 vs. sham	DLPFC vs. Sham
Time to Exhaustion _(Min)_	6.20 ± 1.8	7.54 ± 2.1	8.12 ± 2.7^a^	F_(2,26)=_ 5.27	0.29	0.012	d = 0.67	d = 0.84
CWST _(IG score)_	11.23 ± 5.2	11.27 ± 6.5	14.08 ± 6.7	F_(2,26)=_ 1.38	0.09	0.26	d < 0.01	d = 0.47
CRT _(Milliseconds)_	499.64 ± 78.3	456.85 ± 97.4	412 ± 52.1^a^	F_(2,26)=_ 4.36	0.25	0.023	d = 0.48	d = 1.3
Heart Rate _(Beats Per Min)_	142.2 ± 13.9	138.8 ± 13.1	144.9 ± 13.5	F_(2,26)=_1.01	0.07	0.37	d = 0.25	d = 0.19
O_2_ saturation _(%)_	80.14 ± 3.1	80.50 ± 4.2	82.57 ± 2.9	F_(2,26)=_ 2.44	0.16	0.10	d = 0.11	d = 0.82
EMG of VL _(% of MVC)_	22.36 ± 9.1	25.54 ± 9.1	28.08 ± 8.4	F_(1.3,17.2)=_ 1.95	0.13	0.18	d = 0.35	d = 0.65
EMG of VM _(% of MVC)_	28.92 ± 10.7	34.24 ± 11.9	39.69 ± 10.1^a^	F_(1.3,18.1)=_4.71	0.27	0.03	d = 0.46	d = 1.03
EMG of RF _(% of MVC)_	13.83 ± 2.3	14.36 ± 2.1	15.24 ± 2.1	F_(2,26)=_ 1.28	0.09	0.29	d = 0.21	d = 0.64
Perceived exertion _(0–100)_	99.07 ± 5.9	90.17 ± 17.1	88.94 ± 12.9^a^	F_(2,26)=_3.68	0.22	0.04	d = 0.77	d = 1.07
Affective responses _(-5/+5)_	− 0.55 ± 1.5	0.54 ± 1.5	0.89 ± 1.5^*^	F_(1.3,17.4)=_ 6.44	0.33	0.02	d = 0.68	d = 0.9
Felt arousal _(1–6)_	2.21 ± 0.67	2.66 ± 0.5	2.85 ± 0.6^*^	F_(2,26)=_ 3.7	0.22	0.04	d = 0.71	d = 0.96

Data are presented as mean ± standard deviation

*tDCS* transcranial direct current stimulation; *M1* primary motor cortex; *DLPFC* dorsolateral prefrontal cortex; *EMG* electromyography; *VL* vastus lateralis; *VM* vastus medialis; *RF* rectus femoris; *MVC* maximum voluntary contraction; *CWST* color-word Stroop test; *CRT* choice reaction time

^a^ = significant different from sham (all ps < 0.05)

### Effect of tDCS on endurance performance in hypoxia

**Fig. 1:**
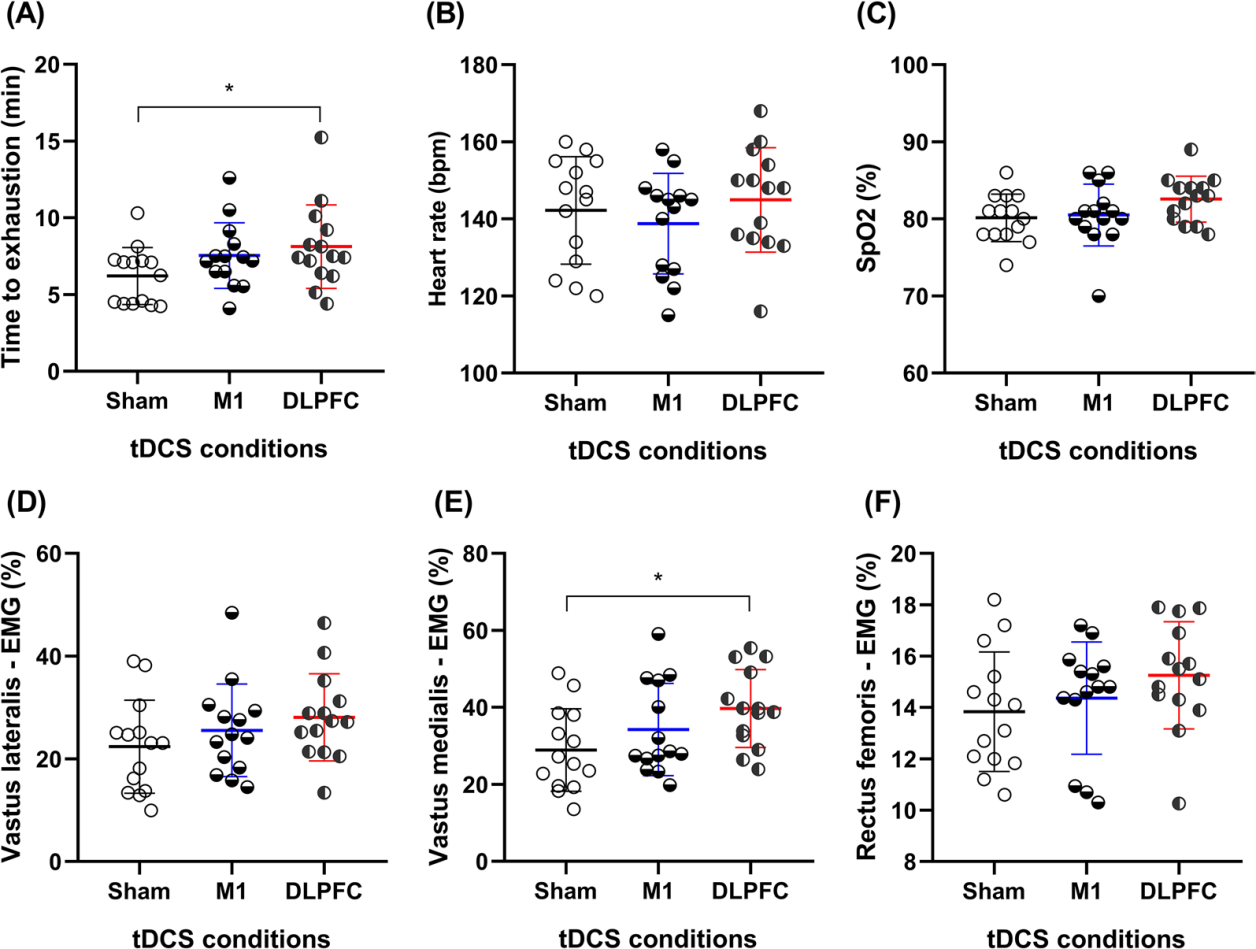
Mean values of TTE, HR, SpO_2_, and EMG under 3 experimental conditions. Endurance **A** cycling time to exhaustion (TTE), **B** heart rate (HR), **C** blood oxygen saturation (SpO_2_), electromyography (EMG) amplitude of **D** the vastus lateralis (VL), **E** vastus medialis (VM), and **F** rectus femoris (RF) muscles under hypoxia, with transcranial direct current stimulation targeting the primary motor cortex (M1), dorsolateral prefrontal cortex (DLPFC), and sham conditions. * = Significantly different from Sham

tDCS over the DLPFC significantly improved (p_=_0.036; Fig. 1A) endurance performance (i.e., longer TTE) compared to sham with a large effect size (d_=_0.84; Δ_=_30.96%). No significant differences were observed in TTE between other conditions (p˃ 0.05).

### Effect of tDCS on physiological responses during the TTE test in hypoxia

tDCS over the DLPFC significantly increased (p_=_0.003; Fig. 1E) **EMG amplitude** of the vastus medialis muscle (VM) compared to sham with a large effect size (d_=_1.03, Δ_=_37.24%). No significant differences were found between other conditions for the EMG amplitude of the VM (p˃0.05). There were no significant differences between tDCS conditions in **EMG amplitude** of the vastus lateralis (VL; Fig. 1D) and rectus femoris muscles (RF; Fig. 1F). Similarly, tDCS did not change **HR** (Fig. 1B) and **SpO**_**2**_ (Fig. 1C) during TTE test.

### Effect of tDCS on psychophysiological responses during the TTE test in hypoxia

**Fig. 2:**
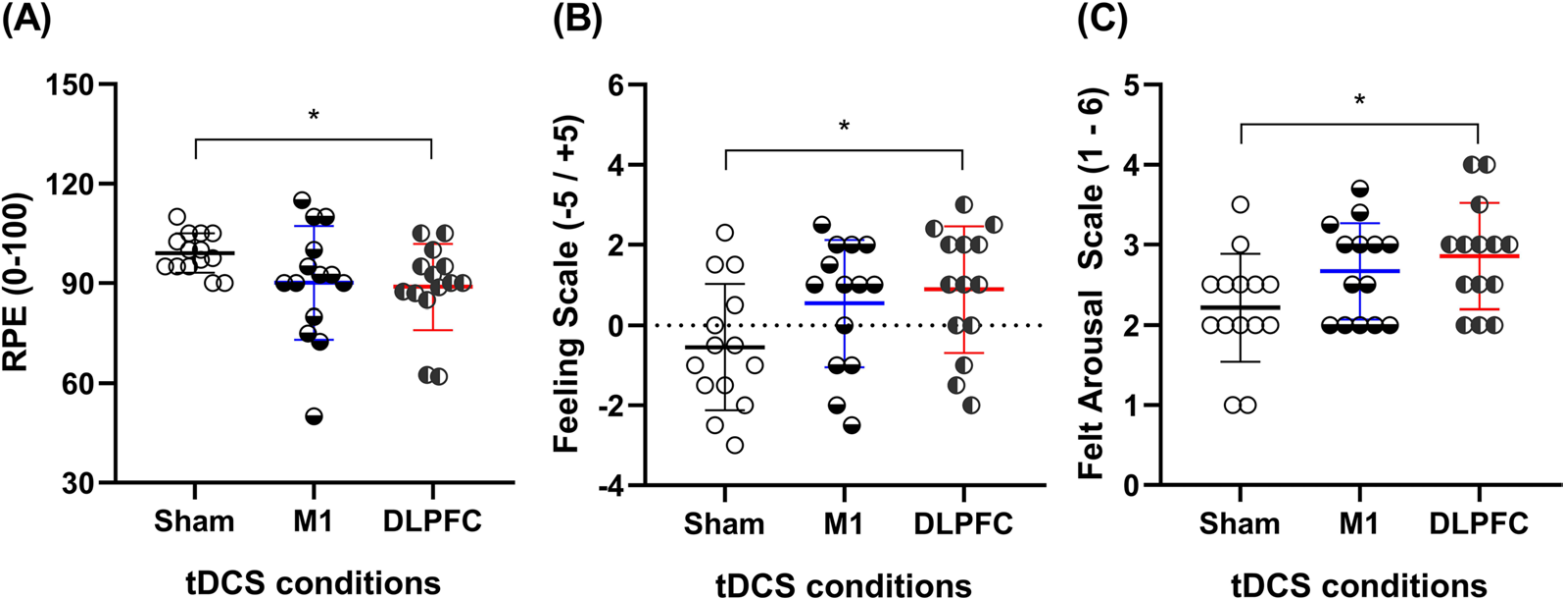
Mean values of RPE, affective responses, and FA under 3 experimental condition. **A** Rating of perceived exertion (RPE), **B** affective response, and **C** felt arousal during the endurance cycling task in hypoxia after transcranial direct current stimulation targeting the motor cortex (M1), dorsolateral prefrontal cortex (DLPFC), and sham conditions. * = Significantly different from Sham

tDCS over the DLPFC significantly decreased **RPE** (p_=_0.045; Fig. 2A) compared to sham with a large effect size (d_=_1.07; Δ_=_10.23%), but no other significant differences were found (p˃0.05). Similarly, tDCS over the DLPFC significantly increased the **affective responses** (p_=_0.035; Fig. 2B) and **FA** (p_=_ 0.029; Fig. 2C) compared to sham, with large effect sizes (d_=_ 0.9, Δ_=_ 260%, and d_=_ 0.96, Δ_=_ 28.9%, respectively), but no other significant differences were found for these variables (p˃0.05).

### Effect of tDCS on cognitive performance after the TTE test in hypoxia

**Fig. 3:**
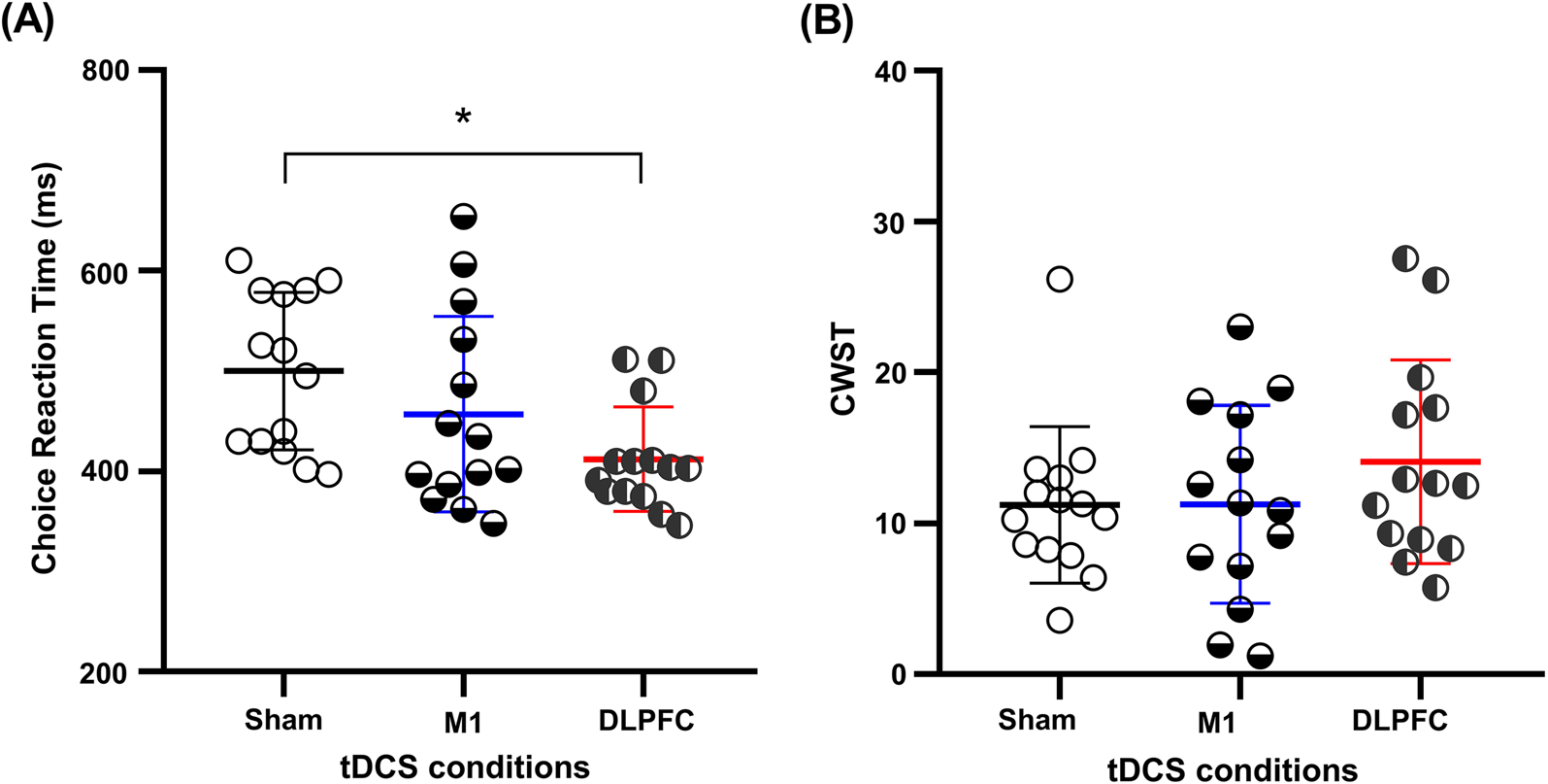
Mean values of CRT and CWST score after endurance exhaustion under 3 experimental conditions. **A** Choice reaction time (CRT) and **B** color-word Stroop test (CWST) scores immediately after endurance time to exhaustion test in hypoxia with transcranial direct current stimulation targeting the motor cortex (M1), dorsolateral prefrontal cortex (DLPFC), and sham conditions. * = Significantly different from Sham

tDCS over the DLPFC significantly decreased post-exhaustion **CRT** (p_=_ 0.01, Fig. 3A) compared to sham with a large effect size (d_=_ 1.3; Δ_=_− 17.55%), but no other significant differences were found (p˃0.05). There was no significant difference in post-exhaustion **CWST** performance among tDCS conditions (Fig. 3B).

### Complementary analysis

The TTE in DLPFC tDCS was negatively correlated with RPE (rho = − 0.57; p = 0.035), but not correlated with VM’s EMG (r = − 0.15; p = 0.62), affective responses (r = 0.27; p = 0.34) or felt arousal (r = 0.19; p = 0.53).

## Discussion

To the best of our knowledge, this work is the first to examine the effects of anodal-tDCS over the M1 and DLPFC areas on endurance performance, cognitive function, and psychophysiological responses under hypoxic conditions. As a novel finding, we observed that the participants had a significantly longer TTE under hypoxia (O_2_ = 13% ⋍ 3500 m altitude) after left DLPFC-tDCS but not M1-tDCS, compared to the sham condition. Interestingly, the longer TTE following left DLPFC tDCS coincided with increased VM muscle EMG amplitude, decreased RPE, and increased affective responses and arousal. However, TTE in the DLPFC condition presented a significant (negative) correlation only with RPE, so the lower RPE the higher TTE. No other correlation with EMG, affective responses, and felt arousal was found.

**Fig. 4:**
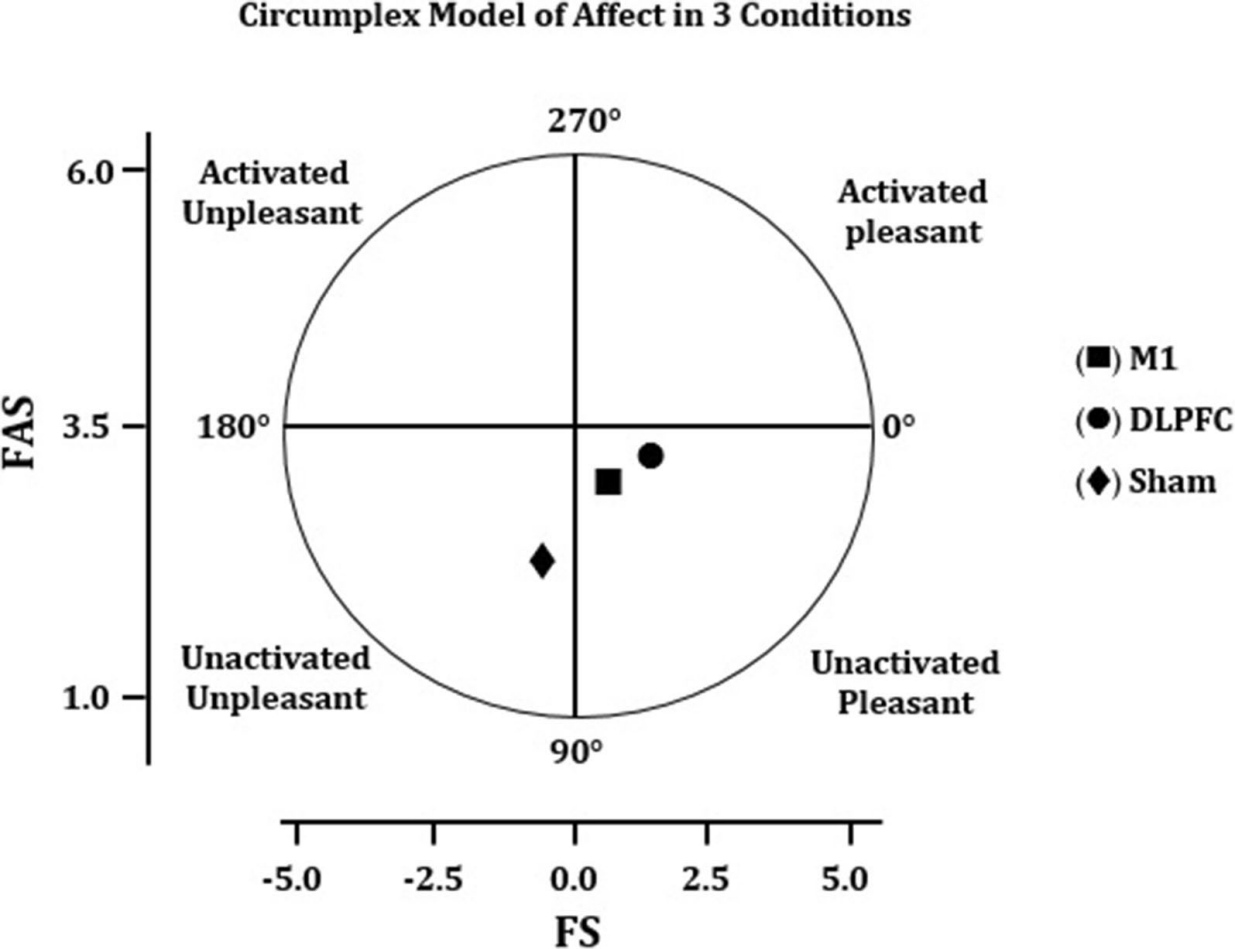
The two-dimension Circumplex Model of Affect under 3 experimental conditions. The mean value of affective response and felt arousal (FA) were used to create a two-dimension circumplex model of affect during the endurance cycling task in hypoxia after transcranial direct current stimulation targeting the motor cortex (M1), dorsolateral prefrontal cortex (DLPFC), and sham conditions. *FS* feeling scale; *FAS* felt arousal scale

It is widely known that hypoxia can negatively impact both cognitive function and physical performance (especially endurance performance) through both central and peripheral processes [28, 29, 31, 33]. These include changes in neurovascular coupling (which is thought to be the most important mechanism by which the brain functions properly), decreased excitatory drives from the brain to the periphery, decreased O_2_ delivery to the mitochondria of working muscles, and increased firing of mechano- and metabo-sensitive group III and IV muscle afferents [32, 33, 36]. These mechanisms are crucial for endurance performance because it has been demonstrated that the capacity to perform an endurance task is highly sensitive to changes in the aforementioned mechanisms even in normoxic conditions [26]. Interestingly, recent research suggests that in hypoxia, particularly severe hypoxia, the brain plays a more important role in regulating endurance performance [31]. Mira et al. [31] used terms like "brain-hypoxic effect" or "hypoxia-sensitive central component of fatigue" in this context to emphasize that in hypoxia, central mechanisms are the primary cause of fatigue and endurance performance deterioration. To support this idea, the Psychobiological Model of fatigue during endurance exercise assumes that RPE and potential motivation are the most important determinants of endurance performance, both of which are associated with central processing in the brain [25]. This could explain the longer TTE in the DLPFC condition under hypoxia in the current study, as our findings showed that participants in the DLPFC condition had lower RPE and higher affective responses and arousal (as has been demonstrated in the Circumplex Model of Affect in Fig. 4), compared to the sham condition. In fact, TTE was negatively correlated with RPE in the DLPFC condition, corroborating our claim.

Previous research has suggested that the PFC, including the DLPFC, ventromedial, and ventrolateral PFC, regulates exercise performance through higher order processing of interoceptive cues (i.e., afferent feedbacks), emotional and psychological drive (e.g., internal and external motivation, RPE, environmental features, among others), and decision making (i.e., increase/decrease pace or stop exercise) [23, 24]. Angius et al. [6] reported that anodal tDCS targeting the left DLPFC improved endurance cycling performance in normoxia with lower RPE. These findings were attributed to increased motivation as a result of increased activation of the left DLPFC, which is in line with the Psychobiological Model of fatigue during endurance exercise [6]. Furthermore, Robertson and Marino [23] proposed that the PFC (particularly its lateral region) would be involved in exercise tolerance and termination, alongside other brain areas such as the anterior cingulate cortex, premotor area, and orbitofrontal cortex, by creating the pathways for interpreting afferent signals coming from various parts of the periphery. In this case, it has been proposed that the PFC plays a significant role in integrating sensory afferent signals and providing appropriate responses hierarchically, thereby overruling inhibitory inputs and maintaining motor output [4, 20].

The DLPFC tDCS condition also produced more positive psychophysiological responses than the sham condition in the current study. It is worth noting that in a hypoxic environment, O_2_ deficiency has an additive effect on exercise-related stressors, which may result in the development of a more unpleasant environment, negatively affecting exercise performance when compared to a normoxic condition [26]. Indeed, previous research has emphasized the role of different emotions in exercise tolerance, such as pleasure-displeasure (known as Affective responses), arousal, motivation, and the sensation of pain [23, 43]. It has been demonstrated that the PFC integrates these emotions to regulate endurance performance. It appears that anodal tDCS targeting the left DLPFC has provided a compensatory effect in the hypoxic condition by improving the function of the left DLPFC area, possibly by increasing the excitability of neural circuits beneath the stimulation site as well as increased oxygenation of this region through increased blood flow [1, 40]. Based on the Psychobiological Model of fatigue during endurance performance, it appears to be a plausible scenario in which better psychophysiological responses such as higher motivation and better affective response delay the critical time point when an exerciser decides to stop endurance exercise, as it has been hypothesized that disengagement from an endurance task is a cognitive decision-making process that may be regulated in the DLPFC area [25].

The current study also found no difference in the amplitude of EMG of the VL and RF muscles during a cycling endurance task under hypoxia between tDCS conditions. In contrast, only after DLPFC tDCS was the amplitude of EMG of the VM muscle significantly higher than sham. The causative effect of tDCS on muscle EMG has been a contentious topic because most studies found no effect of tDCS on muscle EMG [6, 44, 45]. While some recent findings suggest that tDCS may affect muscle EMG [7, 21, 39, 46]. Changes in motor unit recruitment strategies as a result of brain stimulation have been proposed as a mechanism by which tDCS could induce its effect on muscle activity as reflected by EMG [7, 39]. Surprisingly, while anodal tDCS of M1 changed EMG in previous studies, higher EMG amplitude in VM muscle was found in the DLPFC but not in the M1 condition in the current study. It is unclear why the M1 tDCS did not affect EMG amplitude in this study. One possible explanation is that we did not use transcranial magnetic stimulation (TMS) to precisely target the region representing the motor area of the lower limb in M1 [17]. However, in most previous studies, the international 10–20 EEG system has been confirmed as a valid method for stimulating target areas in the brain [9, 15, 20, 21, 46]. In addition, the montage used in the present study has previously been tested and shown to increase corticospinal excitability and endurance performance [47, 48]. Moreover, the computational modeling of the tDCS-induced electrical field showed that the montage used in the present study in fact reached our nominal target, namely the M1 representation of the lower limbs, which also aligns with previous findings [49]. Alternatively, the demanding nature of the hypoxic condition may have played a regulatory role in the M1 response to anodal tDCS. This raises an intriguing question of whether hypoxia alters the contribution of different areas of the brain, such as M1 and PFC, related to the final neural drive to the working muscles. On the other hand, despite the need for additional research to corroborate the causal influence of the PFC on motor unit recruitment strategies and neural drive to the periphery, it appears that anodal tDCS of the left DLPFC, that lies at the top of the motor hierarchy, was able to create a cascade from the PFC to M1, leading to a change in the recruitment and firing frequency of the target motor units, which was reflected in the EMG amplitude of the VM muscle in the current study. While M1 stimulation has not been able to compensate for inhibitory afferent signals from various peripheral regions that have been amplified due to the burden of the hypoxic condition [4, 7, 20, 23, 39].

Our results also showed that despite a higher CWST score in the DLPFC tDCS condition (Δ_=_25.3%) compared to sham, there was no significant difference in CWST score between conditions. However, after exhaustion in the endurance task under hypoxia, the DLPFC tDCS condition had a significantly shorter CRT compared to sham. Recent studies have found mixed results regarding cognitive function while exercising in hypoxia, emphasizing that we are dealing with a more complex scenario than in normoxia [30, 50–52]. It has been demonstrated that cognitive function is compromised as hypoxia severity increases, most likely due to hypoxia-induced impairment in neurovascular coupling, which is the primary mechanism of brain O_2_ delivery [30, 36, 53]. Recent research has shown that moderate exercise can improve cognitive function even in moderate to severe hypoxia, possibly by increasing arousal via noradrenergic and dopaminergic regulation [36, 51, 54, 55]. Hence, it appears plausible that the cognitive function in this situation is determined by the balance between the positive effects of acute exercise and the negative effects of hypoxia. [30]. The incongruent Stroop test is considered a high cognitive effort task that involves detecting interference between two parallel processes, first, deciding while overlooking unrelated information, and second, inhibiting habitual actions [56, 57]. This emphasizes the importance of proper brain function to process information in such incongruent conditions.

In this context, Ochi et al. [58] demonstrated that hypoxia impaired Stroop test performance and reduced executive function. In our study, despite slightly better performance in CWST under the DLPFC condition, it appears that the potential positive and synergistic effects of endurance exercise and brain stimulation were not able to overcome the detrimental effects of neuromuscular fatigue and hypoxic-induced cognitive burden. On the other hand, CRT performance was significantly lower in the DLPFC condition than in the sham condition. This intriguing finding led us to believe that the nature of the cognitive tasks, in terms of the amount of required cognitive efforts to do the task, is another factor influencing the balance between exercise and hypoxia, which in turn determines cognitive function. In this case, the additive effect of anodal tDCS targeting the left DLPFC and endurance exercise likely mitigated the negative effects of hypoxia and the burden of reaching the point of failure in the present study. The findings of Abedanzadeh et al. [56], which demonstrated that anodal stimulation of the left DLPFC could boost information processing speed and attention capacity (reflecting in a reduced reaction time), lend further support to this notion. Alternatively, improved CRT performance after TTE under hypoxia in the DLPFC tDCS condition may indicate that cognitive capacities were preserved, at least in part, with this specific montage, which in turn provided improved cognitive processing of exercise-related stimulus and greater top-down control, ultimately allowing exercise to be maintained for a longer duration. This would provide further credence to the role of the DLPFC and cognitive processing in exercise regulation [23, 24].

Finally, an important consideration regarding the current study's findings is the fact that, contrary to some previous studies, there was no significant positive effect of M1 tDCS on endurance and cognitive performance, and psychophysiological responses to exercise in hypoxia. This is in line with a recent study by Machado et al. [15], who found no significant effect of conventional and high-definition tDCS targeting M1 on physiological and psychophysiological responses, as well as endurance performance in normoxia in endurance-trained athletes. This raises the question of whether environmental stressors influence the response of different brain areas to tDCS. Thus, it is possible to hypothesize that in the hypoxic condition, where the disruption in O_2_ delivery places a physical and cognitive burden on the body, brain areas such as the PFC, which are involved in the cognitive process, may be more responsive to tDCS and play a more important role in regulating the body's capacity to work appropriately than M1. However, further research with a rigorous design is required to shed light on this topic and provide supporting mechanisms to confirm it.

The lack of use of TMS for hot spotting the lower limb representation in the M1, which could provide a more precise target for tDCS, and the lack of neurophysiological/neuroimaging measures that could provide more information about changes in brain activity are the study's limitations. Finally, to the best of our knowledge, this is the first study to investigate the effect of tDCS on a variety of sports-related variables in hypoxia, and as a result, we were confronted with a lack of information regarding the study's design as well as discussing our findings with more mechanistic interpretations. On the other hand, this is the first study not only to assess the effect of tDCS on endurance exercise in hypoxia but also the first to directly compare two tDCS montages targeting different brain regions on whole-body exercise performance. Radel et al. [20] conducted the only previous study to compare tDCS stimulation of two brain regions and found no effect of either M1 or DLPFC tDCS on single elbow flexion isometric contraction sustained to task failure. As a result, the current study contributes to current knowledge and opens up new avenues for future research to test fatigue model predictions as well as the effectiveness of various tDCS protocols in different environmental conditions.

## Conclusion

The current study found that anodal tDCS targeting the left DLPFC, but not M1, provides an ergogenic boost to endurance performance and cognitive function in hypoxic conditions for the first time. Left DLPFC tDCS also increased EMG of the VM, lowered RPE, and increased affective responses and arousal during TTE. TTE performance in the DLPFC condition was (negatively) correlated only with RPE, implying that tDCS-induced performance enhancement is associated with a decrease in this variable. The current findings support the importance of the PFC, particularly the DLPFC, in exercise regulation and suggest that the responsiveness of different brain areas to tDCS may be affected by environmental stressors (in our case hypoxia-related stressors).

## Methods

### General experimental design

**Fig. 5:**
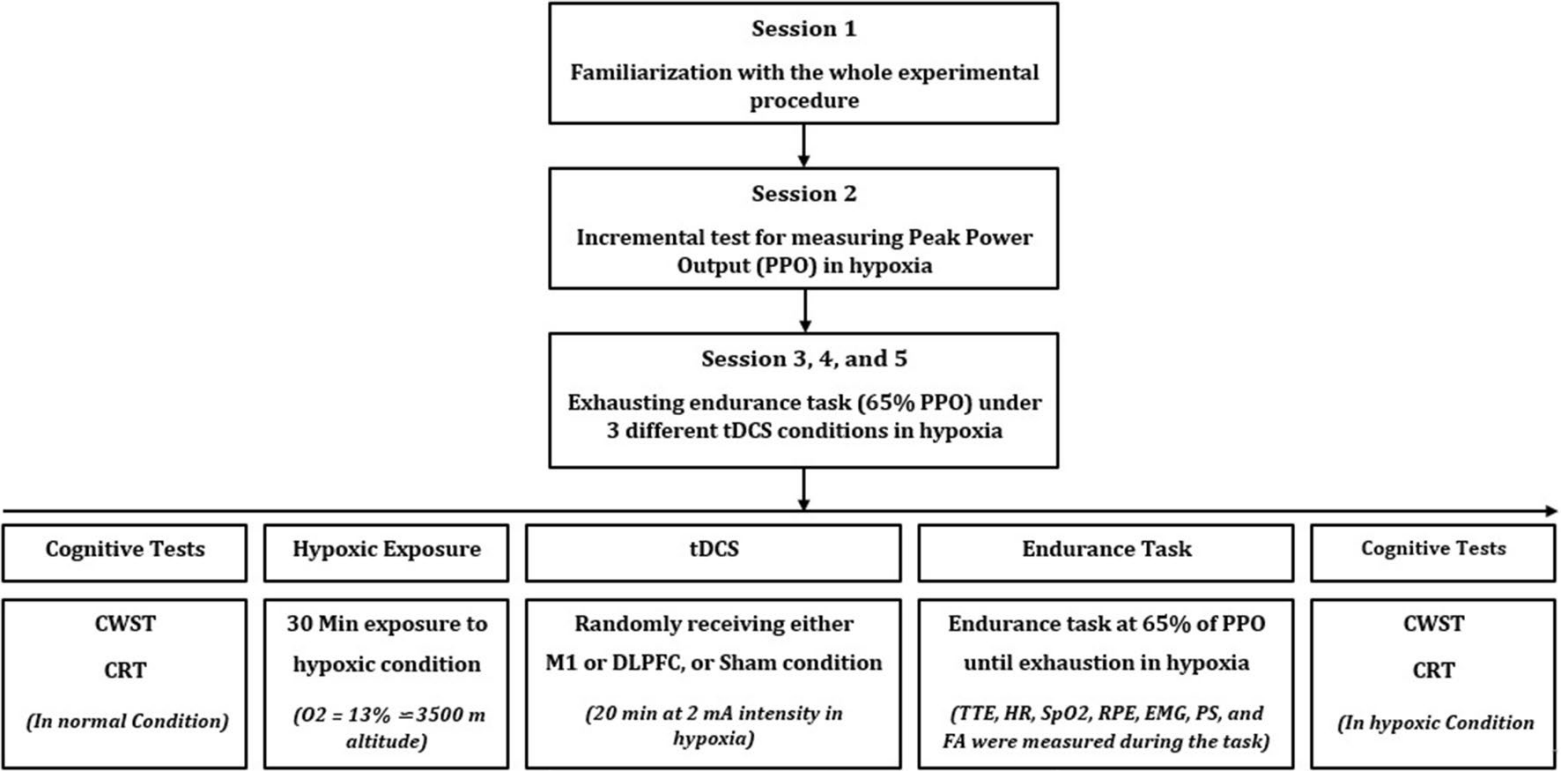
Schematic of the whole study procedure and details of three experimental sessions (M1, DLPFC, and Sham). *PPO* Peak Power Output; *CWST* Color-Word Stroop Test; *CRT* Choice Reaction Time; *tDCS* Transcranial Direct Current Stimulation; *TTE* Time to Exhaustion; *HR* Heart Rate; *SpO2* Blood Oxygen Saturation; *RPE* Rating of Perceived Exertion; *EMG* Electromyography; *PA* Pleasure Sensation *FA* Felt Arousal

To measure the effect of different tDCS montages on our primary outcome measures (TTE, EMG amplitude, and RPE during exercise and Stroop test performance immediately after exercise under hypoxia) and secondary outcome measures (affective responses, arousal, HR, and SpO_2_ during exercise and CRT performance immediately after exercise under hypoxia), participants underwent five experimental sessions interspersed with a one-week interval. The first session was designed for familiarizing the participants with the whole experimental procedure, cycling on the ergometer, hypoxic exposure, brain stimulation, and measuring the study variables. Written informed consent was also obtained from each participant in the first session. In the second session, participants performed an incremental cycling test to measure the peak power output (PPO) after 30 min exposure to the hypoxic condition in a resting position. The PPO was used to adjust the intensity of the endurance task performed by each participant within the next three experimental sessions. Then, the counterbalancing procedure was performed using the Latin Squares method by an individual out of the research team to specify the order of receiving 3 different conditions including (1) M1 anodal-tDCS, (2) DLPFC anodal-tDCS, (3) sham-tDCS. The participants and the research team were blinded regarding the type and site of stimulation in each session (i.e., double-blind design). Afterward, through the 3^rd^ to 5^th^ sessions, participants first performed cognitive tests including CWST and CRT in the normal condition. Then, the maximal isometric voluntary contraction (MIVC) test of knee extensor muscles was performed. The MIVC was used for normalizing the EMG data of target muscles during the endurance task in each session. The participants were then exposed to the hypoxic condition in a resting position (seating on a comfortable chair) by wearing an inflatable air cushion mask and breathing a 13% oxygen gas using a hypoxic gas generator for 30 min followed by 20 min of brain stimulation (M1 or DLPFC, or sham stimulation). Accordingly, each subject was in hypoxic condition for 50 min before performing the endurance task. After the termination of brain stimulation, participants performed an endurance cycling task at 65% of previously measured PPO until voluntary exhaustion under the hypoxic condition. During the task, HR and SpO_2_ were measured every minute and RPE, affective responses, and FA were measured every 3 min. EMG of the rectus femoris (RF), vastus lateralis (VL), and vastus medialis (VM) muscles were also measured during the task. After reaching the point of failure, RPE, affective response, and FA were measured, and then, CWST and CRT tests were performed in the hypoxic condition. A 24-h paper-based dietary recall was applied by a nutrition expert (through an interview with each participant) in the second session and, the participants were instructed to follow the same diet 24 h before the next experimental session. Moreover, to avoid any effects of circadian rhythm on the study variables, each subject came to the laboratory at the identical time of the day in a laboratory-controlled ambient condition (19–22 °C; 50–60% relative humidity) in all experimental sessions. The whole experimental procedure has been depicted in Fig. 5.

## Participants

Fourteen endurance-trained males, naive to tDCS, voluntarily took part in this randomized, counter-balanced, double-blind, and sham-controlled study. The characteristics of the participants are presented in Table 3. The sample size was calculated a priori using G*Power software (Version 3.1.9.2, Kiel, Germany) as follows: test family = F tests; Statistical test = ANOVA: Repeated measures, within factors; α error probability = 0.05; power (1-β err prob) = 0.80; Effect size f = 0.45 (based on the systematic review and meta-analysis by Machado et al. [1] in which the effect of tDCS on exercise performance was investigated), number of groups = 1, number of measurements = 3. Accordingly, 10 participants would be appropriate as the sample size for the present study. Considering the possibility of a relatively high dropout rate considering the number of sessions (crossover design) and its duration [59], 15 participants were recruited for this study. One participant withdrew from the study for personal reasons, and 14 participants completed the whole experimental procedure.

**Table 3:** General characteristics of the participants (n = 14)

Variables	Mean ± SD
Age _(years)_	23.78 ± 4.28
Body mass _(kg)_	76.81 ± 8.9
Height _(cm)_	182.35 ± 6.7
Body mass index _(kg/m_^2^_)_	23.03 ± 1.6
Body fat _(%)_	16.1 ± 3.5
Fat mass _(kg)_	12.55 ± 3.71
Fat-free mass _(kg)_	64.3 ± 6.4
Training sessions _(days/week)_	3.85 ± 0.7
Training duration _(min/day)_	96.42 ± 12.7
Training experience _(years)_	5.35 ± 1.3
Peak power output _(W)_	271.42 ± 46.8

The inclusion criteria were (1) endurance-trained males (at least 3 training sessions per week), (2) no stay in altitude above 2000 m over the last two months, (3) having no blood donation within the last two months, (4) without a history of seizure, epilepsy, or other neurological diseases, (5) having no implanted medical devices or pacemakers in the body, (6) acute and chronic cardiovascular or orthopedic diseases, (7) no tobacco, drug, and alcohol consumption, (8) no poor or deficient color vision. The exclusion criteria were [1] not completing the study protocol or abandoning the study and [2] injury related or not to the study that could influence the outcome variables. All participants gave their written informed consent to the experimental design of the study. All experimental procedures were conducted following the declaration of Helsinki. This study was registered in the Iranian Registry of Clinical Trials (IRCT id: IRCT20210617051606N6; https://www.irct.ir/trial/61519; Registration Date: 17.02.2022). The first participant was included on 02.03.2022, and the trial was terminated on 25.05.2022 in Kermanshah, Iran.

## Peak power output measurement

In the second visit to the laboratory, a maximal incremental test was performed under hypoxia using a cycle ergometer (Cyclus 2, RBM Elektronik-automation GmbH, Leipzig, Germany) by each participant to determine the peak power output (PPO). To do so, each participant first was exposed to hypoxia for 30 min in a resting position and then performed an incremental cycling test until voluntary exhaustion. Before starting the test, the position of the saddle was adjusted for each participant and this position was recorded to be used in the subsequent sessions. The test started at 50 W for 2 min with the pedal cadence of 60 rpm and then, the power output increased by 50 W each 3 min (with the same cadence, 60 rpm) until the volitional exhaustion. Verbal encouragement was applied to avoid test withdrawal before reaching the real point of failure. Participants reported their RPE on a 0–100 Borg scale during the last 10 s of each 3-min phase and at the time of exhaustion. Meeting two of the following criteria during the incremental test was deemed as the point of real exhaustion: (1) HR ≥ 90% of age-predicted maximal HR (220-age), (2) inability to maintain the pedal cadence of 60 rpm for more than 5 s despite verbal encouragement, (3) RPE ≥ 90. The PPO was calculated according to the following formula: *PPO* = *W*_*out*_ + *(t/180)* × *50 [W*_*out*_*: workload of the last completed stage; t: time in the final stage in seconds]* [60]. The PPO was used for adjusting the intensity of the endurance task performed by the participants within the three subsequent sessions.

## Cognitive function measurement

### Color-word stroop test (CWST)

The standardized version of the paper-based color-word Stroop test revised by Golden (1975), consisting of 3 cards listing 100 items each presented in a “5 (columns) × 20 (rows)” matrix was used in the present study. The card I (word; W) included randomly distributed 100 words (red, green, and blue) printed in black ink on a white sheet while no word was followed by itself in a column. Card II (color; C) consists of 100 colors (written as XXXX in color) printed in either red, green, or blue on a white sheet in which no color was followed by itself in a column or matched the corresponding word on card I. This means none of the colors on card II match the position of the words on card I. Finally, card III (color-word; CW) contained 100 colored words on a white sheet in which the order of words from the card I was printed in the order of the colors from card II. This way no word for a color matched that particular color. The participants were given all three cards with card ‘W’ on top as the first card, followed by card ‘C’ as the second one, then card ‘CW’ as the third card, placed in front of them on a flat surface. They were instructed to read out loud as many items in each card in 45 s (45 s per card) as quickly as they could. Accordingly, the participants started the test by reading down the columns of card ‘W’ within 45 s when the experimenter stopped them and circled the last item read by the participants. The same procedure was applied for cards ‘C’ and ‘CW’. If there was a mistake, the experimenter said “No” and the participants had to correct the mistake and continue the test. Moreover, if the participants finished all the columns of each card before 45 s, they were instructed to return to the first column of that card and read again. The number of items correctly named in 45 s in each card was recorded and used to calculate the predicted CW (PCW) score according to the following formula: [PCW = (W × C) / (W + C)]. Then, the PCW score was subtracted from the actual score of the CW card (number of items correctly named in CW card) leading to obtaining the interference score (IG) as follows: IG = CW – PCW [61]. Higher IG scores indicate a greater ability to inhibit interference and better cognitive function. The CWST was performed at the beginning of each experimental session (before hypoxic exposure) and immediately after exhaustion in the endurance task under hypoxia.

### Choice reaction time (CRT)

The Visual Choice Reaction Time Apparatus (Model 63035A, Lafayette Instrument Company, Indiana, USA) was used to measure CRT. A four-choice compatible stimulus–response paradigm was used. The participants sat comfortably in a chair in front of the response panel having four lights and corresponding response buttons beneath each light. Five randomized visual stimuli (lights turning on) were manually given to the participants and they were instructed to respond as quickly as they could by pushing the corresponding button on the response panel. The reaction time in each stimulation was recorded and the mean value of 5 efforts was calculated as each subject’s final score of CRT. The CRT was performed at the beginning of each experimental session (before hypoxic exposure) and immediately after exhaustion in the endurance task under hypoxia.

## MIVC

After performing the cognitive tests in the 3rd to 5th sessions, participants performed 3–5 s knee extension MIVC three times with a 15-s rest in between on a custom-made chair with knee and hip fixed at 90° as recommended for VL, VM, and RF muscles MIVC test [62]. The standard warm-up was carried out before the MIVCs and verbal encouragement was provided during the test. The best performance in MIVCs was recorded for normalizing the EMG signals of that session.

## Hypoxia exposure

The target hypoxic condition (O_2_ = 13%, equivalent to an altitude of ~ 3600 m or 12,000 feet) was induced using a hypoxic air generator equipped with a semipermeable filtration membrane (GO_2_Altitude ERA II, Biomedtech, Melbourne, Australia). The generator constantly pumped the hypoxic air into two 120-L Douglas bags. For inducing the hypoxic condition in each experimental session, participants wore an inflatable air cushion mask with a non-rebreathing valve positioned and fixed comfortably by the use of a rubber port full face harness. The mask was connected to the Douglas bags. Participants then sat in a comfortable chair breathing a gas mixture containing 13% of O_2_ for 50 min. During the last 20 min of hypoxic exposure, tDCS was applied to each participant. Subjects also wore the mask and breathed the 13% O_2_ concentration during the entire TTE test. The mask was removed only after the cognitive testing after exhaustion in the TTE. SpO_2_ was also continuously monitored and recorded using a fingertip pulse oximeter during all experimental sessions.

## Transcranial direct current stimulation (tDCS)

Through the 3^rd^ to 5^th^ experimental sessions and after 30 min of hypoxic exposure in a resting position, participants received one of the three brain stimulation conditions including (1) M1 anodal-tDCS, (2) DLPFC anodal-tDCS, and (3) sham-tDCS in a randomized, counter-balanced and double-blind design for 20 min at 2 mA intensity under hypoxia. A battery-driven stimulator (NeuroStim 2, Medina Tebgostar, Tehran, Iran) was used to apply direct current over the target areas in the brain. Two carbon electrodes (5 × 4 cm; 20 cm^2^) covered by surface sponges soaked in saline solution (NaCl 140 mmol dissolved in Milli-Q water) were used as an anode and cathode. The electrodes were held in place using elastic bands. The international 10–20 EEG system and a 64-channel EEG cap were used to locate target areas over the scalp. Two different stimulation montages were used in this study as follows: (1) M1 montage—the anode symmetrically placed over the Cz (2.5 cm of each side of the M1) covering the representation of the lower limbs motor area and cathode placed over the left shoulder (extra-cephalic cathode); (2) DLPFC montage—the anode placed over F3 representing the left DLPFC area and cathode placed over AF8 representing the supraorbital region. These montages were chosen based on the most recent findings in related studies to have optimal stimulation in each target area [9, 15, 17, 21]. In the M1 montage, we chose an extracephalic montage as it was demonstrated in simulation studies to induce a greater electric field in the motor cortex than bicephalic and High-definition tDCS [49] increase corticospinal excitability, as assessed by motor-evoked potentials [47] and is more effective than a bicephalic montage to improve endurance performance, likely through avoiding the negative effects of the cathode on excitability [48]. Finally, the anode centered at Cz followed the electrode placement in other studies targeting the motor representation of the lower limbs [11, 15, 47, 63].

In M1 and DLPFC conditions, the current was gradually ramped up for 30 s, maintained at 2 mA for 20 min, and then progressively ramped down for 30 s. In the sham condition, the montage was the same as the DLPFC condition, and the same 30 s ramping up and down was used, but the 2-mA current was only maintained for 30 s. This sham protocol has been used in previous studies and has been shown to induce similar sensations as the active tDCS protocol [21, 64, 65]. Moreover, to make sure that the participants were effectively blinded to the stimulation conditions, they were not informed that there is a sham (inactive) stimulation condition. Only after they finished the complete study protocol, they were fully debriefed on the study aims and procedures.

**Fig. 6:**
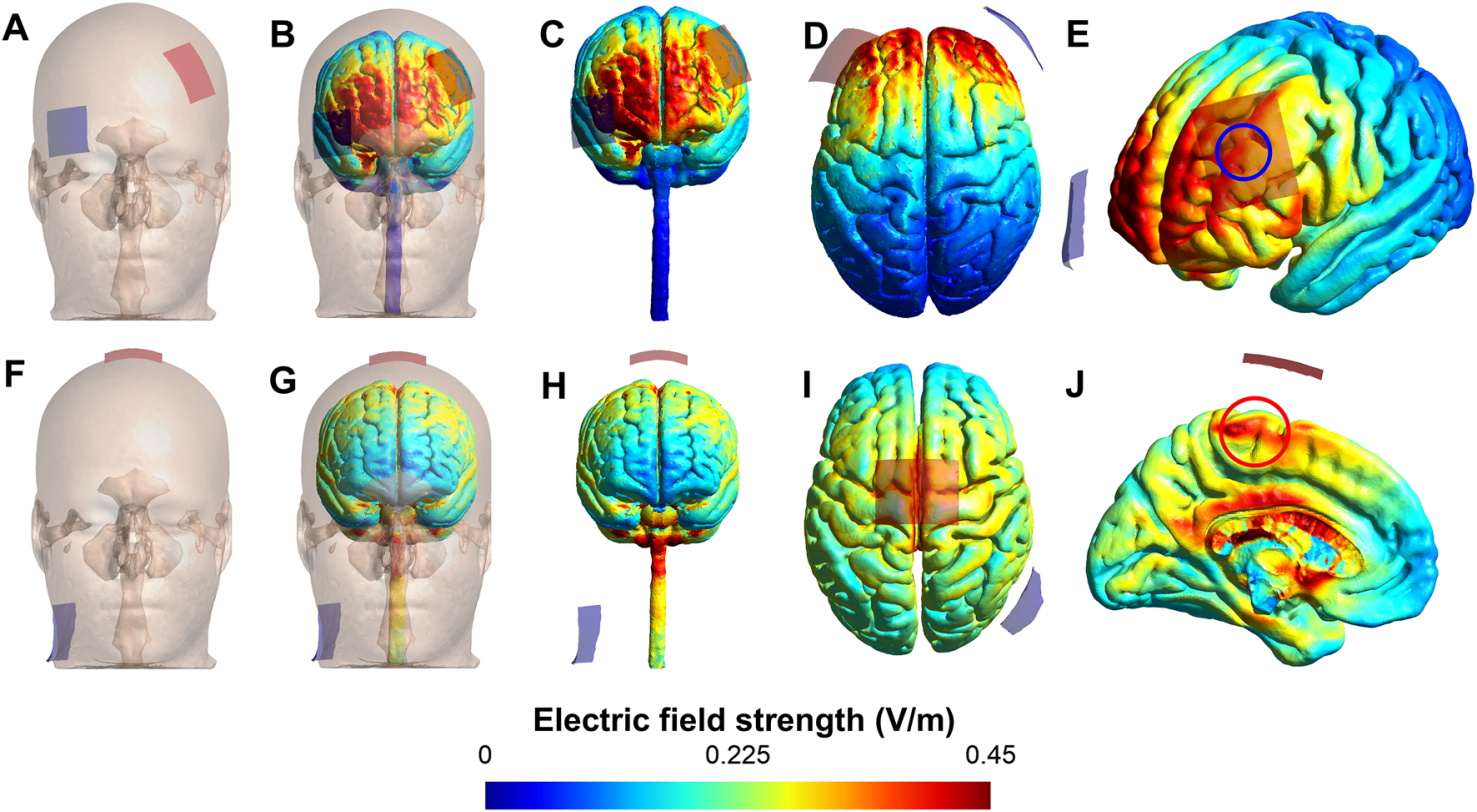
tDCS-induced electric field on brain areas for the study montages. tDCS-induced electric field on brain areas for the montages targeting the left dorsolateral prefrontal cortex (top line) and primary motor cortex representation of the lower limbs (bottom line). Anodal (red rectangle) and cathodal (blue rectangle) electrodes placed over the scalp (**A**, **B**, **F**, and **G**). Figures are color-coded according to the electric field strength so that hot colors (e.g., red) represent stronger electric fields and cold colors (e.g., blue) represent weaker electric fields. Frontal (**C** and **H**) and top (**D** and **I**) view of the electric current distribution in gray and white matter. Diagonal (**E**) and sagittal (**J**) view of the electric current distribution in gray matter with arrows roughly over the nominal targets (blue = left dorsolateral prefrontal cortex; red = primary motor cortex representation of the lower limbs). Note: Because the anatomical model does not include a shoulder for the M1 tDCS montage, the cathode electrode was placed on the lower part of the neck, which provides a good approximation of the should placement

The brain current flow during tDCS was calculated using a finite element model (FEM) following the standard pipeline in SimNIBS 4.0.0 [66]. The magnetic resonance imaging (MRI) MNI 152 head model available in the software was used. MRI data were segmented into surfaces corresponding to the white matter (WM), gray matter (GM), cerebrospinal fluid (CSF), skull, and skin. The electrical conductivities of each segment were determined according to values previously established as follows: WM = 0.126 Siemans/meter (S/m), GM = 0.275 S/m, CSF = 1.654 S/m, bone = 0.010 S/m, and skin/scalp = 0.465 S/m [67], rubber electrode = 29.4 S/m, and saline-soaked sponges = 1.000 S/m. All information concerning the respective tDCS montages was entered into the software: current intensity = 2 mA; electrode position (+ F3/-AF8 and + Cz/-left shoulder); electrode and sponge sizes (5 × 4 cm); electrode thickness = 1 mm; sponge thickness = 5 mm. Because the anatomical model does not include a shoulder for the M1 tDCS montage, the cathode electrode was placed on the lower part of the neck, which provides a good approximation of the should placement. The results of the simulations are presented in Fig. 6. As can be seen in Fig. 6, the montage targeting the DLPFC did reach the target but also other prefrontal areas between the electrodes. Finally, the M1 tDCS reached the motor representation of the lower limbs, also producing large electrical fields in deeper regions of the brain and the spinal cord (Fig. 6).

## tDCS-induced sensations and blinding assessment

Participants completed a questionnaire provided by Fertonani et al. [68] after each session, listing the sensations and level of intensity experienced during the stimulation. Itching, pain, burning, warmth/heat, pitching, metallic/iron taste, fatigue, and other sensations (open questions) were all listed on the questionnaire. The degrees were none (zero), mild (one), moderate (two), considerable (three), and strong [4]. Participants also indicated whether these sensations affected their ability to perform the exercise (0 = not at all; 1 = slightly; 2 = considerably; 3 = much; 4 = very much); when the discomfort started (1 = beginning; 2 = at about the middle; 3 = towards the end); and when it stopped (1 = stopped quickly; 2 = stopped in the middle; 3 = stopped at the end). An aggregate variable (referred to as "discomfort" generated by tDCS) was computed as the total of the strength scores recorded for all sensations so that the discomfort variable ranged from 0 (lack of discomfort) to 28 (maximum discomfort). According to recent literature, the end of the study corrects guess rates, which indicates the percentage of participants that successfully guessed their experimental condition, which might lead to a misleading interpretation of blinding effectiveness [69, 70]. It has been suggested to report the “active stimulation guess rate”, which indicates the percentage of participants who guessed they received the active treatment [69]. Hence, despite we report both correct and active stimulation guess rates, we will consider the latter as the measure of blind effectiveness [69].

## Whole-body endurance task

*TTE in a cycling endurance task:* In sessions 3, 4, and 5, after 30 min exposure to the hypoxic condition in a resting position followed by 20 min of brain stimulation (50 min in total under hypoxia), participants performed a cycling endurance task on a cycle ergometer (Cyclus 2, RBM Elektronik-automation GmbH, Leipzig, Germany) in a hypoxic condition until voluntary exhaustion. They started the task by performing 5 min of warm-up at 45% of the previously measured PPO followed by continuous cycling at power output equivalent to ⋍ 65% of PPO and pedal cadence of 60 rpm until volitional exhaustion. The same saddle position individually chosen for performing the incremental test was applied for each participant. HR and SpO_2_ were measured every minute during the task. RPE, affective responses, and arousal was reported every three minutes and at the end of TTE. Verbal encouragement was provided to each subject throughout the task to avoid test withdrawal before reaching the real point of failure. Observing two of the following criteria during the task was considered the point of real exhaustion: (1) HR ≥ 90% of age-predicted maximal HR (220-age), (2) inability to maintain the pedal cadence of 60 rpm for more than 5 s despite verbal encouragement, (3) RPE ≥ 90 on 0–100 Borg scale.

## Physiological responses

### ***HR and SpO***_***2***_

During the whole experimental session, HR was continuously monitored by the use of a chest strap (M430, Polar, Finland) connected to the cycle ergometer. SpO_2_ was also constantly measured and recorded by a fingertip pulse oximeter (Nonin Onyx II 9550, Nonin Medical, Plymouth, MN, USA).

### EMG

The surface EMG signals were strictly collected according to the recommended standards [71, 72]. In each experimental session, surface wireless EMG sensors (Ultium ™ wireless EMG system, Noraxon, Inc., Scottsdale, AZ, USA) were placed and fixed on the muscle belly of the VL, VM, and RF muscles of the dominant leg after skin preparation (shaving, abrading, and cleaning with alcohol). EMG signals were amplified (× 1.000), high-pass and low-pass filtered (10 and 500 Hz, respectively), and sampled up to 4000 Hz with the common mode rejection ratio of < -100 dB. EMG signals were then registered and analyzed using MyoRESEARCH 3 software (Noraxon, Inc., Scottsdale, AZ, USA) according to the instruction related to EMG amplitude analysis. To do so, EMG signals were normalized to the MIVC measured at the beginning of each session for that same muscles. The mean value of the EMG amplitude of the VL, VM, and RF muscles (normalized to MVC) during the entire endurance cycling task under hypoxia was recorded and used for statistical analyses.

## Psychophysiological responses

### RPE

The 0–100 Borg scale was used to measure RPE. Participants were familiarized with the Borg scale and received instruction on how to rate their perceived exertion at the end of each stage and the point of exhaustion in the incremental test, and every 3 min and upon reaching the point of exhaustion in the endurance cycling task in hypoxia [73]. All these responses were used to calculate average RPE responses for each experimental condition and used for statistical analyses.

### Affective response and arousal (alertness)

The Feeling Scale (FS), a bipolar scale comprising 11 items ranging from − 5 (very bad) to + 5 (very good), developed, and validated by Hardy and Rejeski was used to measure affective response. The positive numbers represent pleasure, the negative numbers represent displeasure, and zero represents a neutral affective valence. Perceived activation (arousal) was measured using the Felt Arousal Scale (FAS) consisting of 6 items ranging from 1 (low arousal) to 6 (high arousal). During the familiarization session, the participants were acquainted with the concept of affective response, arousal, and how to report their perceptual responses while performing the endurance task under the hypoxic condition (at every 3 min during the task and at exhaustion). All these responses were used to calculate average FS and FAS responses for each experimental condition and used for statistical analyses. The Circumplex Model of Affect proposed by Russell et al. (1980), was used to analyze the perceptual responses since it has been reported to be more consistent with many current findings from behavioral, cognitive neuroscience, neuroimaging, and developmental studies of affect. This model contains two interrelated neurophysiological systems including valence (a pleasure-displeasure continuum) and arousal or alertness (an activation-deactivation continuum). Accordingly, each emotion is considered a linear combination of valence and arousal [74].

## Statistical analyses

Values are presented as means and standard deviation (SD) or median and interquartile range (IQR) as stated. The Friedman test was used to compare tDCS-induced sensations, followed by Wilcoxon signed-rank tests conducted with a Bonferroni correction for pair-wise comparisons (0.05/3 = Bonferroni corrected p = 0.017), in case of significant differences. The mean value of TTE, HR, SpO_2_, EMG amplitude for the VL, VM, and RF muscles, RPE, affective responses, and FA during each experimental session was calculated and used for statistical analyses. Concerning cognitive tests, since there were no statistical differences in CWST and CRT performance at baseline among the experimental conditions (under normoxic conditions), only the values of the post-exhaustion under hypoxia were used for statistical analyses.

The normal distribution of each data set was evaluated by the Shapiro–Wilk normality test. One-way repeated measures ANOVA was performed on the mean value of the study variables to analyze the main effect of the condition and when significant, the post hoc test using Bonferroni correction for multiple comparisons was used for the pairwise comparisons [75]. In case of a violation in the assumption of sphericity, the Greenhouse–Geisser epsilon correction was applied. Partial eta squared (*ɳ*^*2*^_*p*_) was used as a measure of the effect size for the ANOVAs and interpreted as small (0.01–0.059), medium (0.06 to 0.139), or large (≥ 0.14). Cohen’s d calculation of the effect size was also used for pairwise comparison and interpreted as small (0.20–0.49), medium (0.50–0.79), or large (≥ 0.80). Finally, we conducted an auxiliary analysis to measure the association between the TTE and the VM’s EMG, affective responses, felt arousal, and RPE using the Pearson and Spearman correlation coefficients in the DLPFC tDCS condition. The significance level for all tests was defined as *p˂0.05*. The statistical analyses were performed using SPSS software, version 23 (SPSS Inc., Chicago, IL, USA).

## Data Availability

The data generated and/or analyzed during the current study are available from the corresponding author or reasonable request.

## Abbreviations

CRT: Choice reaction time
CWST: Color-word Stroop test
DLPFC: Dorsolateral prefrontal cortex
EMG: Electromyography
FA: Felt arousal
M1: Primary motor cortex
MIVC: Maximal isometric voluntary contraction
RF: Rectus femoris
RPE: Rating of perceived exertion
SpO_2_: Blood O_2_ saturation
tDCS: Transcranial direct current stimulation
TTE: Time to exhaustion
VL: Vastus lateralis
VM: Vastus medialis
